# Retraction: A methylene blue assisted electrochemical sensor for determination of drug resistance of *Escherichia coli*

**DOI:** 10.3389/fchem.2025.1631230

**Published:** 2025-05-29

**Authors:** Frontiers Editorial Office

The journal retracts the 31 May 2021 article cited above.

Following publication, the authors contacted the Editorial Office to request the retraction of the cited article, stating that the data in the publication is not reliable. Specifically, the authors identified errors in Figures 6 and 7, involving the misrepresentations of %RA values for bacterial strains. This inconsistency affected the distinction between sensitive and drug-resistant strains using the electrochemical method. These inaccuracies were discovered by the authors analysing the original data during further studies on the same subject. An investigation was conducted in accordance with Frontiers’ policies that confirmed this; therefore, the article has been retracted.

This retraction was approved by the Chief Editors of Frontiers in Chemistry and the Chief Executive Editor of Frontiers. The authors agree to this retraction.

